# Analysis of clinical and methodological characteristics of early COVID-19 treatment clinical trials: so much work, so many lost opportunities

**DOI:** 10.1186/s12874-021-01233-w

**Published:** 2021-02-26

**Authors:** Beatrice Mainoli, Tiago Machado, Gonçalo S. Duarte, Luísa Prada, Nilza Gonçalves, Joaquim J. Ferreira, João Costa

**Affiliations:** 1 Laboratory of Clinical Pharmacology and Therapeutics, Faculdade de Medicina, Universidade de Lisboa, Avenida Prof. Egas Moniz, 1649-028 Lisbon, Portugal; 2 Instituto de Medicina Molecular, Faculdade de Medicina, Universidade de Lisboa, Lisbon, Portugal; 3 Centro de Estudos de Medicina Baseada na Evidência, Faculdade de Medicina, Universidade de Lisboa, Lisbon, Portugal; 4 Nova IMS - Universidade Nova de Lisboa, Lisbon, Portugal; 5 Cochrane Movement Disorders Group, Lisbon, Portugal

**Keywords:** COVID-19, Clinical trials, Meta-research, Trial methodology, Clinical endpoints

## Abstract

**Background:**

The COVID-19 pandemic continues to rage on, and clinical research has been promoted worldwide. We aimed to assess the clinical and methodological characteristics of treatment clinical trials that have been set forth as an early response to the COVID-19 pandemic.

**Methods:**

First, we reviewed all registered clinical trials on COVID-19. The World Health Organization International Trials Registry Platform and national trial registries were searched for COVID-19 trials through April 19th, 2020. For each record, independent researchers extracted interventions, participants, and methodological characteristics.

Second, on September 14th, 2020 we evaluated the recruitment status and availability of the results of COVID-19 treatment trials previously identified.

**Results:**

In April 2020, a total of 580 trials evaluating COVID-19 treatment were registered. Reporting quality was poor (core participant information was missing in 24.1 to 92.7%). Between 54.0 and 93.8% of the trials did not plan to include older people or those with a higher baseline risk. Most studies were randomised (67.9%), single-centre (58.3%), non-industry-funded (81.1%), to be conducted in China (47.6%), with a median duration of 184 days and a median sample size of 100 participants. Core endpoints (mortality, clinical status, and hospitalization length) were planned to be assessed in 5.2 to 13.1% of the trials. Five months later, 66 trials (11.4%) were reported as “Completed”, and only 46 (7.9%) had public results available. One hundred forty-four of 580 trials (24.8%) either had the status “Not yet recruiting” or “Suspended”, and 18 (3.1%) trials were prematurely stopped (“Terminated” or “Withdrawn”) The number of completed trials and trials with results are much lower than anticipated, considering the planned follow-up.

**Conclusions:**

Our results raise concerns about the success of the initial global research effort on COVID-19 treatment. The clinical and methodological characteristics of early COVID-19 treatment trials limit their capability to produce clear answers to critical questions in the shortest possible time.

**Supplementary Information:**

The online version contains supplementary material available at 10.1186/s12874-021-01233-w.

## Background

Since December 2019, SARS-CoV-2 has caused a global outbreak of a respiratory illness termed coronavirus disease (COVID-19). COVID-19 ranges from mild, self-limiting respiratory tract illness to severe progressive pneumonia, multiorgan failure, and death [1, 2]. To date, there are no therapeutic agents specifically designed for the treatment of COVID-19. Fierce medical research is currently underway, however, there are historical reasons that led us to question if the global research community is maximizing the expected benefit from these efforts [3]. To shed some light on this question we decided to assess the clinical and methodological characteristics of treatment clinical trials set forth as an early response to the COVID-19 pandemic.

Previous analyses of COVID-19-related studies have been published focusing on scientific articles on COVID-19, and registered studies beyond clinical trials [4]. We aim to focus on clinical trial records, providing a deeper insight into the methodological characteristics that help to assess the capability of clinical research in a pandemic context. COVID-related clinical trials have already been recognized to lack features that optimize their scientific value [5]. We intend to add a further perspective providing a follow-up of the early-phase research and review the output of early COVID-19 treatment trials.

## Methods

### Registry search and trial selection

The World Health Organization (WHO) International Clinical Trials Registry Platform (ICTRP) contains the trial registration datasets provided by 17 clinical trial registries [6]. It is anticipated that the great majority of ongoing clinical trials are captured and recorded by the databases [7]. We searched the ICTRP for completed and ongoing COVID-19 trial records through April 19th, 2020 using the search terms “COVID-19”, “SARS-Cov-2”, “2019-nCoV”, “severe acute respiratory syndrome coronavirus 2”, “2019 novel coronavirus” and “COVID”. We also searched the three main national clinical trial registries, namely ClinicalTrials.gov, the EU Clinical Trials Register (EUCTR), and the Chinese Clinical Trial Registry (ChiCTR).

We included all interventional trials, irrespective of the intervention under investigation. We excluded duplicate trial entries and trials that did not directly address COVID-19. We did not exclude trials due to incomplete data reporting.

### Data extraction

Independent authors selected the trials registered up to April 19th, 2020, extracted data into a pre-piloted spreadsheet (supplementary material), and classified each trial as seeking to assess a treatment or prophylactic effect, or both. For each record, we extracted the type of intervention, methodological aspects of the study design, and participant characteristics. We assessed whether or not trials plan to include participants with known risk factors for poorer outcomes in COVID-19 [1, 2], namely cancer, chronic obstructive pulmonary disease (COPD), diabetes, heart disease, hypertension, or immunodeficiency.

For each trial identified, we extracted the updated recruitment status on September 14th, 2020, alongside the reasons for trials being prematurely stopped when available. We also assessed the registries for any submitted results or indexed publications on the same date. Additionally, we checked for publications of results of the previously identified trials in WHO’s Global Research on Covid-19 Database [8], Cochrane Covid-19 Study Register [9], and selected living systematic reviews on covid-19 [10, 11].

### Analysis

We conducted statistical analyses using the R software (version 3.6.1). We calculated descriptive statistics to characterize data. We performed statistical comparisons between post hoc defined groups using the Chi-Squared and Kruskal-Wallis tests. We deemed *P* values of 0.05 statistically significant, with tests being two-sided.

### Sample size estimates

We conducted sample size calculations using different baseline scenarios for case fatality rates (CFR) for the control group (2, 5, and 10%), as the true CFR is largely unknown [12] and liable to change over time. Nevertheless, we recognize that a 2% CFR may be lower than that observed in settings recruiting mostly high-risk patients. We considered different possible treatment effects. All sample size calculations considered a significance level of 0.05 and a power of 80%.

## Results

We found 693 records of clinical trial protocols (supplementary material), 648 (93.5%) on COVID-19, and 45 (6.5%) on COVID-related conditions (notably pulmonary rehabilitation or exercise and mental health among health care workers).

Among COVID-19 trials, 572 (88.3%) evaluated treatment only (Table 1), 68 (10.5%) evaluated prophylaxis only, and eight (1.2%) evaluated both. Our results focus on the 580 trials evaluating COVID-19 treatment.

**Table 1:** Trials for COVID-19 treatment

	Pharmacotherapy	Traditional Chinese Medicine	Mesenchymal stem cells and NK cells	Advanced life-support strategies	Convalescent plasma and immunoglobulins	Others
Number of trials – no. (%)	349 (60)	92 (16)	39 (7)	27 (5)	35 (6)	44 (8)
Maximal inclusion age – no. (%)						
≥ 65	115 (33)	56 (61)	26 (67)	10 (37)	12 (34)	17 (39)
≥ 80	61 (17)	26 (28)	7 (18)	9 (33)	6 (17)	6 (14)
Not specified or unknown^a^	228 (65)	32 (35)	12 (31)	17 (63)	20 (57)	27 (61)
Inclusion of severe COVID-19 – no. (%)						
Yes	174 (50)	23 (25)	28 (72)	16 (59)	22 (63)	16 (36)
No	86 (25)	30 (33)	2 (5)	10 (37)	4 (11)	14 (32)
No information	89 (26)	39 (42)	9 (23)	1 (4)	9 (26)	14 (32)
Inclusion of critical COVID-19 – no. (%)						
Yes	70 (20)	3 (3)	12 (31)	17 (63)	16 (46)	8 (18)
No	165 (47)	48 (52)	9 (23)	6 (22)	9 (26)	22 (50)
No information	114 (33)	41 (45)	18 (46)	4 (15)	10 (29)	14 (32)
Inclusion of participants with cancer – no. (%)						
Yes	15 (4)	3 (3)	0 (0)	0 (0)	0 (0)	1 (2)
No	49 (14)	31 (34)	27 (69)	3 (11)	4 (11)	11 (25)
No information	285 (82)	58 (63)	12 (31)	24 (89)	31 (89)	32 (73)
Inclusion of participants with COPD – no. (%)						
Yes	16 (5)	3 (3)	0 (0)	1 (4)	0 (0)	0 (0)
No	31 (9)	31 (34)	7 (18)	3 (11)	3 (9)	6 (14)
No information	302 (87)	58 (63)	32 (82)	23 (85)	32 (91)	38 (86)
Inclusion of participants with diabetes – no. (%)						
Yes	16 (5)	0 (0)	0 (0)	1 (4)	0 (0)	0 (0)
No	16 (5)	11 (12)	2 (5)	3 (11)	1 (3)	6 (14)
No information	317 (91)	81 (88)	37 (95)	23 (85)	34 (97)	38 (86)
Inclusion of participants with heart disease – no. (%)						
Yes	30 (9)	0 (0)	0 (0)	1 (4)	0 (0)	1 (2)
No	52 (15)	23 (25)	3 (8)	1 (4)	4 (11)	5 (11)
No information	267 (77)	69 (75)	36 (92)	25 (93)	31 (89)	38 (86)
Inclusion of participants with hypertension – no. (%)						
Yes	19 (5)	2 (2)	0 (0)	1 (4)	0 (0)	0 (0)
No	8 (2)	4 (4)	0 (0)	1 (4)	0 (0)	1 (2)
No information	322 (92)	86 (93)	39 (100)	25 (93)	35 (100)	43 (98)
Inclusion of immunocompromised participants – no. (%)						
Yes	9 (3)	0 (0)	0 (0)	0 (0)	0 (0)	0 (0)
No	82 (23)	27 (29)	13 (33)	2 (7)	5 (14)	9 (20)
No information	258 (74)	65 (71)	26 (67)	25 (93)	30 (86)	35 (80)
Main geographical locations – no. (%)	China 110 (32) Single European country 103 (30) United States 47 (13)	China 90 (98) Single African country 1 (1) No information 1 (1)	China 28 (72) Single European country 6 (15) Single American country 2 (5)	China 10 (37) Single European country 9 (33) United States 6 (22)	China 11 (31) Single European country 9 (26) United States 6 (17)	China 21 (48) United States 8 (18) Single European country 7 (16)
Primary endpoints used – no. (%)						
Mortality	43 (13)	7 (8)	3 (8)	8 (30)	9 (26)	5 (11)
Clinical status (WHO Scales)	38 (11)	0 (0)	0 (0)	0 (0)	2 (6)	3 (7)
Length of hospitalization	14 (4)	8 (9)	2 (5)	2 (7)	2 (6)	3 (7)
Randomised trials – no. (%)						
Yes	275 (79)	49 (53)	19 (49)	13 (48)	16 (46)	26 (59)
No	74 (21)	43 (47)	20 (51)	14 (52)	19 (54)	18 (41)
Multicentre trials – no. (%)						
Yes	129 (37)	37 (40)	11 (28)	6 (22)	13 (37)	15 (34)
No	164 (47)	47 (51)	26 (67)	17 (63)	16 (46)	25 (57)
No information	56 (16)	8 (9)	2 (5)	4 (15)	6 (17)	4 (9)
Industry-funded – no. (%)						
Yes	73 (21)	12 (13)	9 (23)	2 (7)	6 (17)	7 (16)
No	276 (79)	80 (87)	30 (77)	25 (93)	29 (83)	37 (84)
Median sample size calculated (IQR) – no.	123.5 (60–333)	120 (72–300)	30 (20–48)	44 (20–190.5)	50 (20–117.5)	70 (40–200)
Median expected trial duration (IQR) – days	181 (98.5–365)	155 (90–337)	314 (188–437.5)	217 (92–396)	214 (92–364)	262.5 (90–381.75)

COPD chronic obstructive pulmonary disease, IQR interquartile range

Note: The sum of the columns is higher than 580 because some trials assess more than one intervention

^a^Most of these trials (93%) specify a minimal inclusion age, without specifying a maximal inclusion age

### Quality of reporting

The quality of reporting across the trial registries is globally poor. In trial protocols covering COVID-19 treatment, the maximal inclusion age was not specified in 332 protocols (57.2%), while 159 (27.4%) and 197 (34.0%) failed to report whether participants with severe or critical disease forms were to be included, respectively. Additionally, we could not assess whether patients with known risk factors for poorer outcomes would be included in 74.8% (regarding the inclusion or exclusion of patients with an immunocompromised state) and 93.8% (regarding the inclusion or exclusion of participants with hypertension) of the protocols.

### COVID-19 treatment trials interventions

Most trials (349 of 580, 60.2%) studied pharmacotherapy (drug medicines), though 92 (15.9%) studied traditional Chinese medicine (TCM). The remaining trials (25.0%) evaluated mesenchymal stem cells and natural killer cells, advanced life support strategies, convalescent plasma and immunoglobulins, and other interventions (Table 1).

**Fig. 1:**
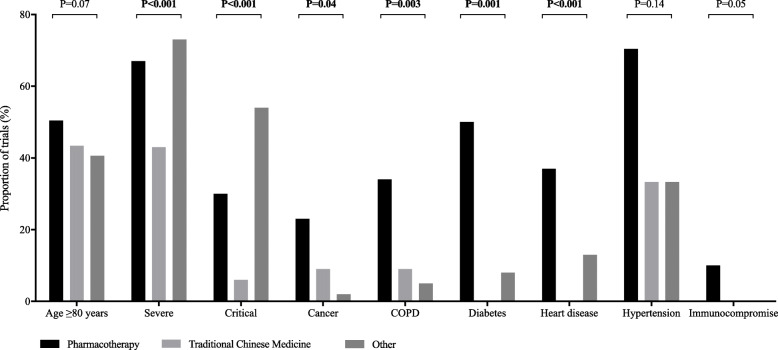
Clinical characteristics of participants included in early COVID-19 treatment trials

Those protocols for which clinical data were available, less than half had planned to include participants over the age of 80 (19.7%), patients at a critical stage (21.4%), and patients with known risk factors for poorer outcomes (range, 1.6 to 5.5%). When comparing pharmacotherapy, TCM, and trials evaluating other treatment interventions, we found notable differences in the proportion of trials that planned to include patients with a severe (*P* < 0.001) or critical status (*P* < 0.001) at baseline, both were smaller among trials evaluating TCM (Fig. 1).

**Fig. 2:**
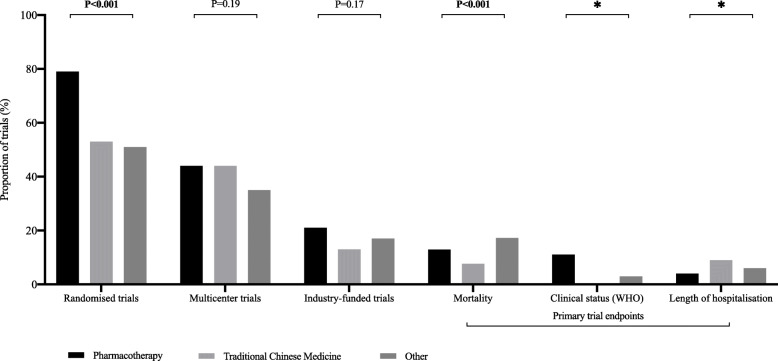
Methodological characteristics of early COVID-19 treatment trials

**Fig. 3:**
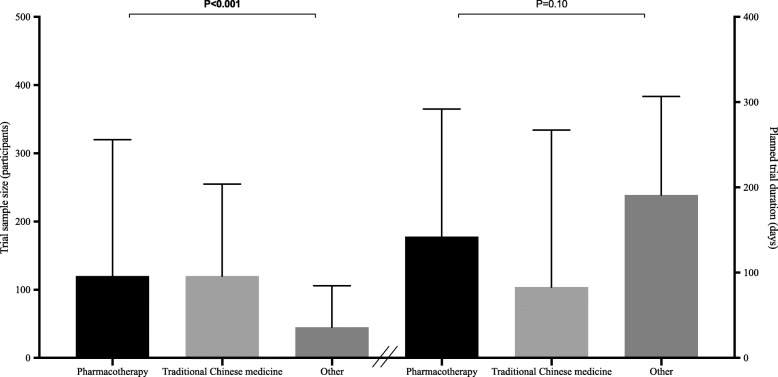
Planned median sample size and trial duration of early COVID-19 treatment trials

In those protocols for which methodological data were available, most trials were non-industry-funded (81.1%), used a randomised design (67.9%), and were conducted in China (47.6%), irrespective of the intervention under evaluation. Less than half the trials (41.7%) planned to have more than one centre and only 3.0% planned to include centres from countries on different continents (Fig. 2). Core clinical outcomes (mortality, clinical status evaluated with WHO scales, and length of hospitalization) were assessed as primary endpoints in only a minority of trials (range, 5.2 to 13.1%). The most commonly reported primary endpoints were respiratory measures (97, 20.8%). The planned median sample size and trial duration were 100 participants (interquartile range, 50 to 260) and 184 days (interquartile range, 94 to 365), respectively. When comparing pharmacotherapy, TCM, and trials evaluating other treatment interventions, we found notable differences in the proportion of trials that were randomised controlled trials (RCTs) and in planned median sample sizes (Fig. 3). Pharmacotherapy trials had the highest proportion of RCTs (*P* < 0.001), while trials evaluating other treatment interventions had the lowest planned median sample sizes (*P* < 0.001).

### Pharmacotherapy trials for COVID-19 treatment

The clinical and methodological characteristics of these trials are detailed in the supplementary material.

Of the 349 trials that sought to evaluate drug treatments for COVID-19, most (111, 31.8%) evaluated chloroquine/hydroxychloroquine. Additionally, 89 (25.5%) evaluated antivirals, 65 (18.6%) assessed monoclonal antibodies, and 42 (12.0%) assessed a form of interferon, immunomodulators, or immunosuppressants. Notably, the proportion of trials that included patients with a severe or critical illness at baseline were different between these groups (*P* < 0.001 for both): the inclusion of these populations was greater among trials evaluating monoclonal antibodies (89.5 and 45.8%, respectively) and smaller among trials evaluating antivirals (43.9 and 13.2%, respectively). The proportion of trials including patients with COPD, diabetes, cancer, or immunodeficiency was also different across these treatment groups, with the inclusion of these populations greater in chloroquine/hydroxychloroquine trials and smaller in antiviral trials (table and figure in the supplementary material). The planned median trial sample sizes and study durations were also larger in chloroquine/hydroxychloroquine trials and smaller in antiviral trials (table and figure in the supplementary material).

### Sample size estimates

We performed sample size calculations for different baseline scenarios for CFR of 2, 5, and 10% in the control group while considering different possible treatment effects on mortality. Regarding registered trials with mortality as a primary endpoint, and assuming a baseline risk of 2%, only three trials were sufficiently powered to detect a difference of 50% or more between treatment groups (supplementary material).

### Availability of results and recruitment status in September 2020

On September 14th, 2020 we assessed the recruitment status and availability of results of the 580 treatment trial protocols registered up to April 19th, 2020. Sixty-six (11.4%) trials were reported as “Completed”, 351 (60.5%) as “Recruiting”, 130 (22.4%) as “Not yet recruiting”, 14 (2.4%) as “Suspended”, 11 (1.9%) as “Terminated”, seven (1.2%) as “Withdrawn”, and for one (0.2%) the recruitment status was unknown.

Of the 18 trials that stopped prematurely (classified as “Terminated” or “Withdrawn”) we collected the reasons for stopping if this was explicit in the registries: six were due to low accrual, three were due to availability of new evidence, two for others reason (including, for example, administrative issues), and seven trials for unknown reasons. Fourteen trials were reported as “Suspended” for the following reasons: six due to low accrual, one due to futility, one due to availability of new evidence, three for other reasons, and three for unknown reasons.

Of the 580 identified treatment trials, 251 (43.3%) had been planned to be completed before September 14th, 2020; nevertheless only 33 of these (13.1%) reported being “Completed” on this date. The majority of these trials remained listed as “Recruiting” (136; 54.2%), 66 (26.3%) as “Not yet recruiting”, six (2.4%) as “Suspended”, seven (2.8%) as “Terminated”, two (0.8%) as “Withdrawn”, and one (0.4%) was unknown.

Public results were only available for a few trials (46, 7.9%), mainly through journal articles, and only one had submitted results to the registry. Of these 46 trials, 19 were listed as “Completed” in the registry and two as “Terminated”, while 22 were still listed as “Recruiting”, and three as “Not yet recruiting”.

## Discussion

On January 30th, 2020, COVID-19 was declared a public health emergency of international concern and on March 11th, it was declared a pandemic. As of our initial search date, April 19th, 1,603,209 cases had been reported worldwide, 54,225 patients were in a serious or critical state, and 169,750 had died [13]. These numbers continued to increase hourly as the world raced to mitigate the impact of the disease. Our results suggest that a considerable clinical research effort was mobilized early on against COVID-19. The hundreds of trials conducted worldwide offer some hope. To maximise the efficacy of research in infectious disease epidemics, research must be fast, flexible, and integrated with the frontline response [14].

Adaptive clinical trials may be particularly useful in the current pandemic [15, 16] as they enable sample sizes and allocation ratios to be refined, treatments or doses abandoned, and focus moved towards patients with a higher likelihood of benefit. A few large trials, notably, the RECOVERY trial and the WHO Solidarity trial, are underway and use an adaptive design. Currently, preliminary results are available confirming the usefulness of this type of trial design in providing informative evidence, namely showing positive results for dexamethasone [17] and lack of benefit for hydroxychloroquine [18] and lopinavir/ritonavir [10]. Another example is the Adaptive COVID-19 Treatment Trial, (ACTT) [19], sponsored by the National Institute of Allergy and Infectious Diseases (NIAID) that led remdesivir to receive a conditional marketing authorisation by the European Medicines Agency (EMA) and an initial Emergency Use Authorization (EUA) followed by formal approval by the US Food and Drug Administration (FDA). With these few exceptions, the research we found seems largely insufficient to provide clear answers for core outcomes in the shortest possible time. However, it is important to consider that although limitations were present from the onset, several protocols were later revised based on the evolution of the pandemic and increasing knowledge of the disease. The overall impact of protocol modifications is beyond the objectives of the present study. Nevertheless, based on the final results of several studies, most limitations present from the onset seem to have remained and affect the final publication.

Our results suggest that patients at the largest risk of death due to COVID-19 were not being prioritised in clinical trials. This is an important missed opportunity since the high baseline risk means that smaller treatment effects would be more easily detected [3]. Therefore, participants with a higher likelihood of benefit should not only be the focus of clinical research from an ethical point of view but would be the most efficient population to study in order to identify which treatments are worthwhile pursuing and which are not. Although the focus on high-risk patients would have been an important strategy to adopt in early trials, in later trials the inclusion of non-selective patients would be essential to ensure the external validity of results.

While most treatment trials use a randomised controlled design, we found that 92 (15.9%) were single-arm trials. These trials are a matter of concern, namely as CFR changes considerably over time, and trials using historical controls will likely lead to more false-positive findings [20]. Also of concern is the fact that most treatment trials (58.3%) were not multicentric. It is well documented that evidence from single-centre trials is more prone to bias compared with multicentre trials, and tend to provide larger treatment effects [21]. This is particularly clear in the critical care setting, where many positive single-centre trials have been contradicted by subsequent multicentre trials [22]. Furthermore, few RCTs conducted in intensive care units and using mortality as a primary endpoint show a beneficial impact of the intervention on the survival of critically ill patients [23].

Another essential aspect of trial design is the choice of endpoints. While we recognize the importance of surrogate endpoints, which allow faster results to be obtained when compared with core clinical endpoints [24], the lack of hard and more pragmatic endpoints such as death and length of hospitalisation are causes for concern. To eschew these more clinically relevant endpoints is a methodological mistake that is hard to understand. High-quality information, as straightforward and generalisable as possible, will be key to moving forward. For research to permit informed clinical decision-making, this will have to change, and trials must use uniform disease-related definitions. Of note, several core outcome sets have been defined since the trial protocols were first registered [25]. However, these were published after the first wave of the pandemic in the northern hemisphere, and therefore beyond the scope of time during which these might have been most useful. Nevertheless, many treatment trials are a priori already deemed not to detect statistically significant and clinically relevant results. The median target sample size of assessed COVID-19 pharmacotherapy trials is 100 participants. This is manifestly insufficient to detect anything but an extremely large treatment effect. For example, regarding trials with mortality as a primary endpoint, only three trials were powered to detect a difference of 50% or more between treatment groups (assuming a baseline risk of 2%). Even assuming a baseline risk of 10%, only 38 trials were adequately powered to detect a difference of 50% or more between treatment groups. In fact, half of the trials evaluating mortality have a sample size of 112 participants or fewer and are only able to identify a treatment effect on mortality over 90% (irrespectively of the baseline risk), which is unrealistic (supplementary material). Although the results of underpowered trials can be meta-analysed, thereby increasing the power to detect a treatment effect, this approach remains inefficient in terms of time and overall resources. The results of systematic reviews and meta-analysis can be threatened by underpowered trials due to publication bias and related types of small-study effects [26]. On the other hand, the planned trial duration is also relatively short (median of 184 days for overall treatment trials), given the uncertainty regarding the natural history of COVID-19.

Some of our concerns have been increased by the early termination of a clinical trial studying remdesivir, conducted in 10 hospitals in Wuhan, China [27]. This trial was to enrol 453 participants but as the disease in the area was brought under control the number of eligible patients became too small, and recruitment was stopped at 236 participants. This led to reduced power in the trial, of only 58%, while it was intended to be 80%. This trial failed to demonstrate any difference in time to clinical improvement with remdesivir (HR 1.23 [95% CI 0.87–1.75]), while, later on, the ACTT trial, an adaptive, larger, and adequately powered trial succeeded in showing the usefulness of remdesivir in hospitalized patients (rate ratio for recovery 1.32 [95% CI 1.12–1.55]) [19].

We found that only 18.9% of treatment trials were industry-funded. Although trials undertaken on the initiative of investigators are important, the absence of experienced industry-supported trials means that there is considerable room for improvement and effort by many of these multinational corporations. Although we are aware of important efforts by the industry to maintain pharmacotherapies available during the crisis, these entities have the resources and experience available to provide an important contribution in conducting high-quality, high-output clinical research.

In mid-March 2020, the majority of COVID-19 cases worldwide were no longer from continental China, and as of April 19th, 2020, only around 4% of cases were from China [13]. These figures are in clear contrast with the fact that so many treatment trials (47.6%) were conducted exclusively in China. Previous research has suggested that a large proportion of clinical trial data submitted to support new drug registrations in China may be considered to be incomplete or substandard [28]. Therefore, despite the large push in research, we question if the upcoming flood of data is of high enough quality to produce clear answers to critical questions at early stages and the necessary recruitment capability. We are also worried that considerable efforts were employed in the 92 treatment trials and combined planned target samples of 18,892 participants studying the effect of TCM on COVID-19. We would urge that these resources be used more wisely.

The main pharmacotherapies under investigation were chloroquine/hydroxychloroquine, antivirals, notably lopinavir/ritonavir, and monoclonal antibodies. We found no substantive methodological differences between trials evaluating these interventions. However, there are differences in patients’ clinical characteristics, with monoclonal antibody trials allowing the inclusion of more patients with several and critical illness, as well as participants with relevant comorbidities. Antiviral trials include relatively fewer patients with several and critical illness, and largely excluded participants with COVID-relevant comorbidities.

As of September 14th, 2020, 6 months after the pandemic was declared, only a minority of these early trials made results available to the public. Additionally, according to planned completion dates, 251 trials should have been completed by this date, while only 33 accomplished this commitment. This corresponds to much lower figures than anticipated, considering the trials’ planned follow-up. Moreover, the information on the recruitment status of the trials often seems to not be updated in the registries, rendering it difficult to interpret the real state of research on this topic. The current available overall results and status from these early 580 treatment trials reinforce our initial worries about the overall inadequacy of these trials to provide clinically relevant conclusions.

Our study has several limitations. First, the data presented focus exclusively on registered trials. Similarly, phase 1 trials may be underrepresented. Second, there is a significant amount of missing or unsubmitted data for certain data fields, which limits the completeness of the analyses and thus the interpretability of the results presented. Third, given the rush to conduct more research, there may be trials underway that had not yet been registered, an important issue given the common nature of retrospective registration [7]. Fourthly, the quality of the available records was largely poor, with inconsistencies and errors throughout. We think that most of the issues have been resolved, though we cannot be certain that nothing was missed.

## Conclusions

With the hundreds of trials enrolling thousands of people currently underway, a more efficient and useful approach would be for research bodies such as the WHO, NIH, Inserm, etc. to create a coordinated research response to face the pandemic. The EMA has called for similar efforts [29]. We understand that there are political, ethical, administrative, contractual, regulatory, logistic, economic, and societal factors that may hinder research, though these difficulties should be overcome in times of global crisis. Persisting on the path of isolated investigations will likely only lead to futile trials and more death on a global scale. Given the large numbers of people with COVID-19, and the recent push for more real-world evidence, we consider that there is an urgent need for a global high-quality COVID-19 patient registry, which could be used to detect large beneficial effects [30] and provide relevant evidence for health care decision-making [31].

Initiatives such as the WHO R&D Blueprint aim to tackle the challenge of generating new evidence during disease outbreaks. We believe that clinical research must be integrated as an essential element of a coordinated international response to epidemics. As those are exceptionally difficult contexts for clinical research, tools such as adaptive protocols that could feasibly be integrated into clinical practice, as well as global research networks and platforms, may be of great help to produce informative research. Due to the unpredictable features of new outbreaks, continued enrolment throughout different locations should be advocated, this would enable the inclusion of sufficient numbers of participants as well as the combination of research efforts [32].

The current treatment and prophylaxis options are few and built upon very scarce and fragile data. With the proper forward planning, critical questions could have been answered earlier. As clinical investigators, we have the obligation to adjust and improve the research being conducted. The world was not ready to react with the appropriate research to a pandemic. We believe that the scientific community, the pharmaceutical industry, and research agencies could have done better.

## Supplementary Information

**Additional file 1: Table S1.** Main pharmacotherapy trials for covid-19 treatment (*n* = 349). **Table S2.** Sample size calculations for mortality. **Figure S1.** Flow chart for trial protocol records. **Figure S2.** Pharmacotherapy trials for covid-19 treatment: clinical characteristics. **Figure S3.** Pharmacotherapy trials for covid-19 treatment: methodological characteristics.

## Data Availability

All data generated or analysed during this study are included in this published article and its supplementary information files.

## Abbreviations

ACTT: Adaptive COVID-19 Treatment Trial
CFR: Case Fatality Rates
ChiCTR: Chinese Clinical Trial Registry
COPD: Chronic Obstructive Pulmonary Disease
COVID-19: Coronavirus Disease 2019
EMA: European Medicines Agency
EUA: Emergency Use Authorization
EUCTR: European Clinical Trials Register
FDA: Food and Drug Administration
ICTRP: International Clinical Trials Registry Platform
IQR: interquartile range
NIAID: National Institute of Allergy and Infectious Diseases
RCT: Randomized Controlled Trials
TCM: Traditional Chinese Medicine
WHO: World Health Organization
