# Supplementary material for: Analysis of clinical and methodological characteristics of early COVID-19 treatment clinical trials: so much work, so many lost opportunities
Source: BMC Med Res Methodol. 2021 Feb 26;21:42. doi: 10.1186/s12874-021-01233-w (PMC7908009; doi:10.1186/s12874-021-01233-w)
Supplement: Supplementary file 1 — Additional file 1: Table S1. Main pharmacotherapy trials for covid-19 treatment (n = 349). Table S2. Sample size calculations for mortality. Figure S1. Flow chart for trial protocol records. Figure S2. Pharmacotherapy trials for covid-19 treatment: clinical characteristics. Figure S3. Pharmacotherapy trials for covid-19 treatment: methodological characteristics. [file 12874_2021_1233_MOESM1_ESM.docx]

**SUPPLEMENTARY MATERIAL**

Early COVID-19 treatment clinical trials: so much work, so many lost opportunities.

Authors: Beatrice Mainoli, MD, Tiago Machado, MD, Gonçalo S Duarte, MD, Luísa Prada, MD, Nilza Gonçalves, MSc, Joaquim J Ferreira, PhD, João Costa, PhD.

**Corresponding author:** João Costa, MD, PhD

Laboratório de Farmacologia Clínica e Terapêutica, Faculdade de Medicina da Universidade de Lisboa, Av. Prof. Egas Moniz, Lisboa, 1649-028, Portugal.

**E-mail:** jncosta@fm.ul.pt
**Phone number:** (+351) 21 797 34 53; **Fax number:** (+351) 21 781 96 88.

**Table of Contents**

[Supplementary Tables: 1](#_Toc63934356)

[Supplementary Table 1: Main pharmacotherapy trials for covid-19 treatment (n = 349) 1](#_Toc63934357)

[Supplementary Table 2: Sample size calculations for mortality 4](#_Toc63934358)

[Supplementary Figures 5](#_Toc63934359)

[Supplementary Figure 1. Flow chart for trial protocol records 5](#_Toc63934360)

[Supplementary Figure 2. Pharmacotherapy trials for covid-19 treatment: clinical characteristics 6](#_Toc63934361)

[Supplementary Figure 3. Pharmacotherapy trials for covid-19 treatment: methodological characteristics 7](#_Toc63934362)

# Supplementary Tables:

## Supplementary Table 1: Main pharmacotherapy trials for covid-19 treatment (n = 349)

|  | Chloroquine or hydroxychloroquine | Antivirals | Monoclonal antibodies | Antimicrobials | Interferons immunomodulators and immunosuppressors | Corticosteroids | ACEI/ARBs | Kinase Inhibitors | Anti-inflammatories | Other |
| --- | --- | --- | --- | --- | --- | --- | --- | --- | --- | --- |
| Number of trials – no. (%) | 111 (32) | 89 (25) | 65 (19) | 42 (12) | 42 (12) | 27 (8) | 16 (5) | 13 (4) | 12 (3) | 62 (18) |
| Maximal inclusion age – no. (%) |  |  |  |  |  |  |  |  |  |  |
| ≥65 | 27 (24) | 38 (43) | 23 (35) | 8 (19) | 19 (45) | 5 (19) | 1 (6) | 4 (31) | 1 (8) | 24 (39) |
| ≥80 | 17 (15) | 14 (16) | 16 (25) | 5 (12) | 5 (12) | 4 (15) | 1 (6) | 3 (23) | 1 (8) | 15 (24) |
| Not specified or unknown * | 80 (72) | 50 (56) | 41 (63) | 32 (76) | 22 (52) | 22 (81) | 15 (94) | 8 (62) | 11 (92) | 38 (6) |
| Inclusion of severe covid-19 – no. (%) |  |  |  |  |  |  |  |  |  |  |
| Yes | 37 (33) | 25 (28) | 51 (78) | 12 (29) | 18 (43) | 18 (67) | 5 (31) | 8 (62) | 6 (50) | 38 (6) |
| No | 36 (32) | 32 (36) | 6 (9) | 11 (26) | 10 (24) | 4 (15) | 3 (19) | 2 (15) | 4 (33) | 13 (21) |
| No information | 38 (34) | 32 (36) | 8 (12) | 19 (45) | 14 (33) | 5 (18) | 8 (50) | 3 (23) | 2 (17) | 11 (18) |
| Inclusion of critical covid-19 – no. (%) |  |  |  |  |  |  |  |  |  |  |
| Yes | 16 (14) | 7 (8) | 22 (34) | 7 (17) | 5 (12) | 12 (44) | 2 (12) | 1 (8) | 1 (8) | 18 (29) |
| No | 56 (50) | 46 (52) | 26 (40) | 21 (50) | 16 (36) | 7 (26) | 6 (38) | 6 (46) | 7 (58) | 27 (44) |
| No information | 39 (35) | 36 (40) | 17 (26) | 14 (33) | 21 (50) | 8 (30) | 8 (50) | 6 (46) | 4 (33) | 17 (27) |
| Inclusion of people with cancer – no. (%) |  |  |  |  |  |  |  |  |  |  |
| Yes | 7 (6) | 1 (1) | 3 (5) | 2 (5) | 1 (2) | 1 (4) | 0 (0) | 1 (8) | 1 (8) | 4 (6) |
| No | 6 (5) | 10 (11) | 17 (26) | 4 (10) | 6 (14) | 3 (11) | 1 (6) | 4 (31) | 1 (8) | 10 (16) |
| No information | 98 (88) | 78 (88) | 45 (69) | 36 (86) | 35 (83) | 23 (85) | 15 (94) | 8 (61) | 10 (83) | 48 (77) |
| Inclusion of people with COPD – no. (%) |  |  |  |  |  |  |  |  |  |  |
| Yes | 9 (8) | 0 (0) | 3 (5) | 1 (2) | 1 (2) | 0 (0) | 0 (0) | 0 (0) | 2 (17) | 3 (5) |
| No | 6 (5) | 7 (8) | 8 (12) | 2 (5) | 6 (14) | 1 (4) | 0 (0) | 1 (8) | 1 (8) | 9 (15) |
| No information | 96 (86) | 82 (92) | 54 (83) | 39 (93) | 35 (83) | 26 (96) | 16 (100) | 12 (92) | 9 (75) | 50 (81) |
| Inclusion of people with diabetes – no. (%) |  |  |  |  |  |  |  |  |  |  |
| Yes | 9 (8) | 0 (0) | 1 (2) | 2 (5) | 1 (2) | 0 (0) | 0 (0) | 0 (0) | 2 (17) | 4 (6) |
| No | 4 (4) | 5 (6) | 0 (0) | 1 (2) | 5 (12) | 1 (4) | 0 (0) | 1 (8) | 0 (0) | 4 (6) |
| No information | 98 (88) | 84 (94) | 64 (98) | 39 (93) | 36 (86) | 26 (96) | 16 (100) | 12 (92) | 10 (83) | 54 (87) |
| Inclusion of people with heart disease – no. (%) |  |  |  |  |  |  |  |  |  |  |
| Yes | 14 (13) | 2 (2) | 5 (8) | 2 (5) | 2 (5) | 0 (0) | 1 (6) | 3 (23) | 2 (17) | 5 (8) |
| No | 17 (15) | 15 (17) | 8 (12) | 4 (10) | 12 (29) | 0 (0) | 0 (0) | 1 (8) | 3 (25) | 9 (15) |
| No information | 80 (72) | 72 (81) | 52 (80) | 36 (86) | 28 (66) | 27 (100) | 15 (94) | 9 (69) | 7 (58) | 48 (77) |
| Inclusion of people with hypertension – no. (%) |  |  | ´ |  |  |  |  |  |  |  |
| Yes | 9 (8) | 1 (1) | 3 (5) | 1 (2) | 2 (5) | 0 (0) | 2 (12) | 1 (8) | 2 (17) | 2 (3) |
| No | 1 (1) | 2 (2) | 2 (3) | 1 (2) | 2 (5) | 0 (0) | 0 (0) | 0 (0) | 0 (0) | 3 (5) |
| No information | 101 (91) | 86 (97) | 60 (92) | 40 (96) | 38 (90) | 27 (100) | 14 (88) | 12 (92) | 10 (83) | 57 (92) |
| Inclusion of immunocompromised people– no. (%) |  |  |  |  |  |  |  |  |  |  |
| Yes | 8 (7) | 1 (1) | 0 (0) | 1 (2) | 0 (0) | 0 (0) | 0 (0) | 0 (0) | 1 (8) | 1 (2) |
| No | 14 (13) | 16 (18) | 34 (52) | 5 (12) | 8 (19) | 11 (41) | 1 (6) | 5 (38) | 2 (17) | 12 (19) |
| No information | 89 (80) | 72 (81) | 31 (48) | 36 (86) | 34 (81) | 16 (59) | 15 (94) | 8 (62) | 9 (75) | 49 (79) |
| Main geographical locations – no. (%) | Single country – Europe 39 (35)  United States 18 (16)  China 17 (15) | China 46 (52)  Single country – Asia 12 (13)  Single country Europe 12 (13) | Single country – Europe 32 (49)  China 14 (22)  United States 11 (17) | Single country -Europe 14 (33)  United States 10 (24)  Single country – America 7 (17) | China 21 (50)  Single country Asia 8 (19)  Single country – Europe 8 (19) | Single country – Europe 14 (52)  China 6 (22)  Single country Asia 3 (11) | Single country – Europe 7 (44)  United States 7 (44) | Single country Europe 8 (62)  Single country – America 2 (15)  United States 2 (15) | Single country – Europe 7 (58)  Global 2 (17) | China 22 (35)  Single country - Europe 16 (26)  United States 8 (13) |
| Primary endpoints used – no. (%) |  |  |  |  |  |  |  |  |  |  |
| Mortality | 18 (16) | 10 (11) | 9 (14) | 7 (17) | 6 (14) | 9 (33) | 2 (12) | 0 (0) | 3 (25) | 9 (15) |
| Clinical status (WHO scales) | 12 (11) | 10 (11) | 10 (15) | 5 (12) | 6 (14) | 0 (0) | 1 (6) | 3 (23) | 2 (17) | 9 (15) |
| Length of hospitalization | 7 (6) | 5 (6) | 1 (2) | 2 (5) | 0 (0) | 0 (0) | 0 (0) | 0 (0) | 1 (8) | 4 (6) |
| Randomised trials – no. (%) |  |  |  |  |  |  |  |  |  |  |
| Yes | 89 (80) | 69 (78) | 52 (80) | 33 (79) | 34 (81) | 26 (96) | 15 (94) | 7 (54) | 10 (83) | 49 (79) |
| No | 22 (20) | 20 (22) | 13 (20) | 9 (21) | 8 (19) | 1 (4) | 1 (6) | 6 (46) | 2 (17) | 13 (21) |
| Multicentre trials – no. (%) |  |  |  |  |  |  |  |  |  |  |
| Yes | 52 (47) | 35 (39) | 26 (40) | 17 (41) | 16 (38) | 10 (37) | 6 (37) | 3 (23) | 2 (17) | 23 (37) |
| No | 43 (39) | 48 (54) | 25 (38) | 14 (33) | 23 (55) | 16 (59) | 7 (44) | 6 (46) | 5 (42) | 28 (45) |
| No information | 16 (14) | 6 (7) | 14 (22) | 11 (26) | 3 (7) | 1 (4) | 3 (19) | 4 (31) | 5 (42) | 11 (18) |
| Industry-funded – no. (%) |  |  |  |  |  |  |  |  |  |  |
| Yes | 14 (13) | 19 (21) | 20 (31) | 7 (17) | 4 (10) | 2 (7) | 1 (6) | 2 (15) | 0 (0) | 18 (29) |
| No | 97 (87) | 70 (79) | 45 (69) | 35 (83) | 38 (90) | 25 (93) | 15 (94) | 11 (85) | 12 (100) | 44 (71) |
| Median sample size calculated (IQR) – no.** | 250 (100-510) | 108 (60-400) | 134 (60-276) | 258 (86-500) | 90 (40-290) | 141 (100-400) | 500 (152-651) | 94 (64-386) | 156 (100-584) | 100 (60-200) |
| Median expected trial duration (IQR) – days*** | 259 (108-457) | 151 (75-327) | 189 (92-351) | 253 (115-457) | 131 (74-305) | 145 (78-214) | 277 (220-381) | 177 (91-286) | 187 (121-214) | 198 (122-304) |

COPD, chronic obstructive pulmonary disease; IQR, interquartile range.

Note: The sum of the columns is higher than 349 because some trials assess more than one intervention.

* Most of these trials (96%) specify a minimal inclusion age, without specifying a maximal inclusion age.

** P<0.001 for comparison between antivirals, chloroquine/hydroxychloroquine, monoclonal antibodies, and others.

*** P<0.05 for comparison between antivirals, chloroquine/hydroxychloroquine, monoclonal antibodies, and others

## Supplementary Table 2: Sample size calculations for mortality

##

| **Proportion of death** | | | | | |
| --- | --- | --- | --- | --- | --- |
| **Example** | **P1 - Active** | **P2 - Control** | **Relative Risk Reduction** | **Relative Risk** | **Sample in each group** |
| 1 | 0.02% | 2% | 99% | 0.01 | 399 |
| 2 | 0.1% | 2% | 95% | 0.05 | 450 |
| 3 | 0.2% | 2% | 90% | 0.1 | 525 |
| 4 | 0.4% | 2% | 80% | 0.2 | 725 |
| 5 | 0.6% | 2% | 70% | 0.3 | 1026 |
| 6 | 0.8% | 2% | 60% | 0.4 | 1503 |
| 7 | 1.0% | 2% | 50% | 0.5 | 2318 |
| 8 | 1.2% | 2% | 40% | 0.6 | 3860 |
| 9 | 1.4% | 2% | 30% | 0.7 | 7285 |
| 10 | 1.6% | 2% | 20% | 0.8 | 17340 |
| 11 | 1.8% | 2% | 10% | 0.9 | 73146 |
|  | | | | | |
| 1 | 0.5% | 5% | 90% | 0.1 | 206 |
| 2 | 1.0% | 5% | 80% | 0.2 | 284 |
| 3 | 1.5% | 5% | 70% | 0.3 | 401 |
| 4 | 2.0% | 5% | 60% | 0.4 | 587 |
| 5 | 2.5% | 5% | 50% | 0.5 | 905 |
| 6 | 3.0% | 5% | 40% | 0.6 | 1505 |
| 7 | 3.5% | 5% | 30% | 0.7 | 2837 |
|  | | | | | |
| 1 | 1.0% | 10% | 90% | 0.1 | 99 |
| 2 | 2.0% | 10% | 80% | 0.2 | 137 |
| 3 | 3.0% | 10% | 70% | 0.3 | 193 |
| 4 | 4.0% | 10% | 60% | 0.4 | 282 |
| 5 | 5.0% | 10% | 50% | 0.5 | 434 |
| 6 | 6.0% | 10% | 40% | 0.6 | 720 |
| 7 | 7.0% | 10% | 30% | 0.7 | 1355 |

# Supplementary Figures

## Supplementary Figure 1. Flow chart for trial protocol records

Protocols identified through ICTRP searching
(n = 909)

Additional protocols identified through other sources
(n = 297)

Trial protocols included in analysis
(n = 693)

Protocols after duplicates removed
(n = 1158)

Protocols screened
(n = 805)

Observational studies protocols excluded
(n = 353)

Trial protocols excluded, with reasons
(n = 112)

Cancelled (n = 37)

Outbreaks related studies (n =47)

Screening/diagnostic trials (n = 16)

Transmission prevention studies (n = 12)

## Supplementary Figure 2. Pharmacotherapy trials for covid-19 treatment: clinical characteristics

##


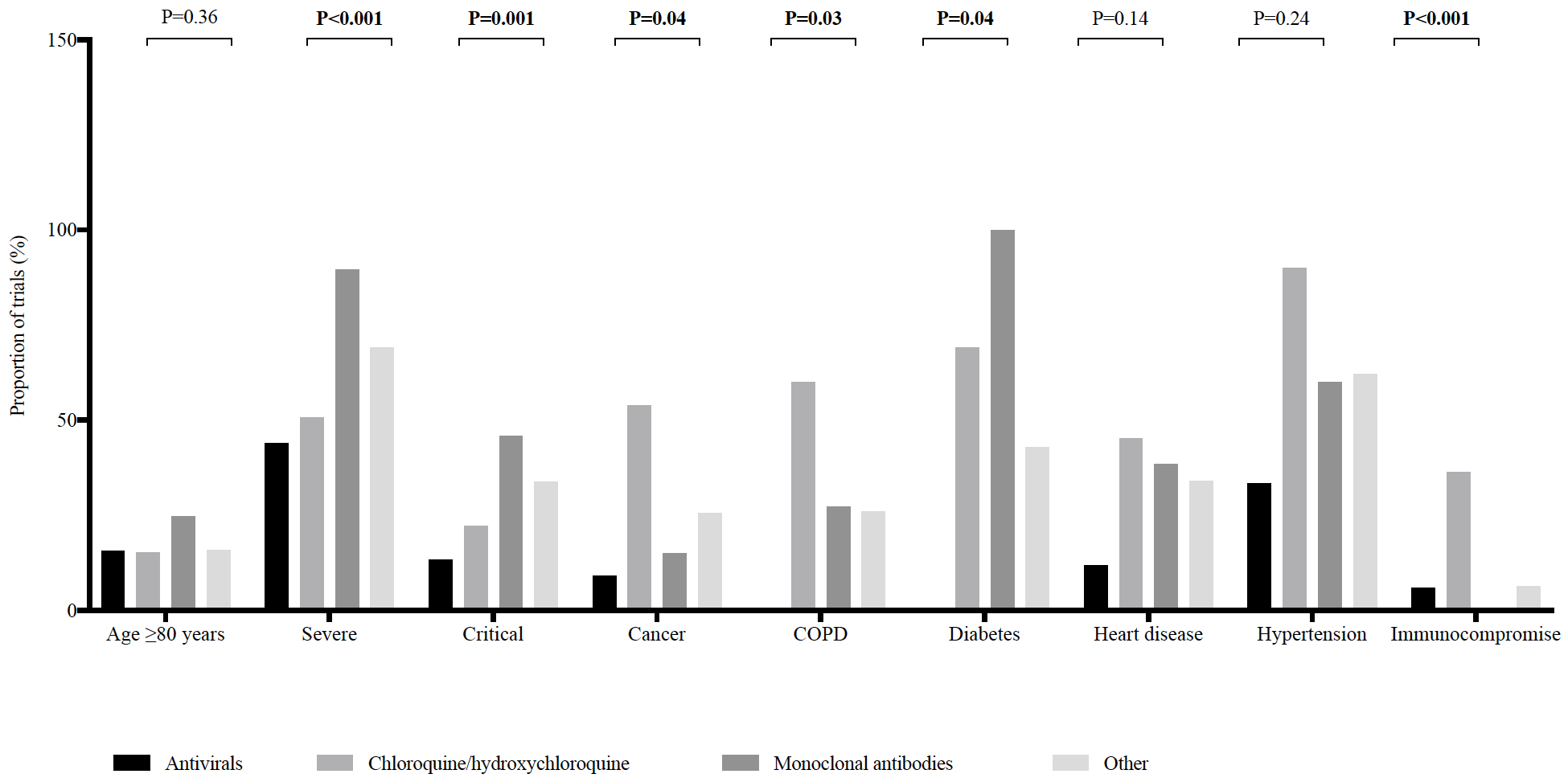


## Supplementary Figure 3. Pharmacotherapy trials for covid-19 treatment: methodological characteristics


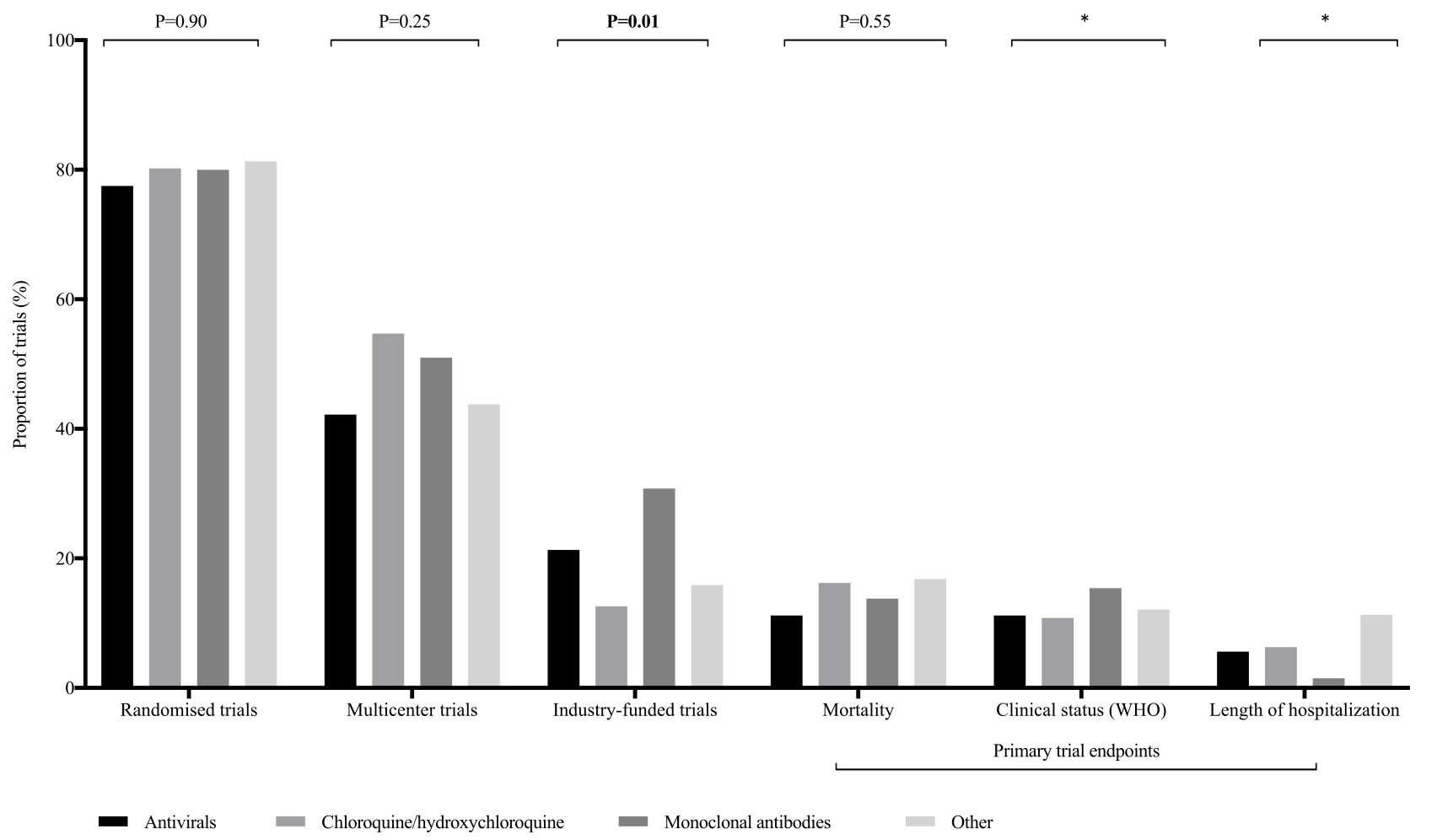


* No formal statistical analysis was conducted due to data sparsity.
